# Risk Factors for the Failure of Spinal Burst Fractures Treated Conservatively According to the Thoracolumbar Injury Classification and Severity Score (TLICS): A Retrospective Cohort Trial

**DOI:** 10.1371/journal.pone.0135735

**Published:** 2015-08-18

**Authors:** Jieliang Shen, Linfei Xu, Baolong Zhang, Zhenming Hu

**Affiliations:** 1 Department of Orthopaedic Surgery, the First Affiliated Hospital of Chongqing Medical University, 1 Youyi Rd., Chongqing, 400016, China; 2 Department of Orthopaedic Surgery, The People's Hospital Of Zhengzhou, No.33 Huanghe Rd, Zhengzhou, Henan Province, 450003, China; University of Michigan, UNITED STATES

## Abstract

**Background:**

The management of thoracolumbar (TL) burst fractures is still controversial. The thoracolumbar injury classification and severity score (TLICS) algorithm is now widely used to guide clinical decision making, however, in clinical practice, we come to realize that TLICS also has its limitations for treating patients with total scores less than 4, for which conservative treatment may not be optimal in all cases.

**Purpose:**

The aim of this study is to identify several risk factors for the failure of conservative treatment of TL burst fractures according to TLICS algorithm.

**Methods:**

From June 2008 to December 2013, a cohort of 129 patients with T10-l2 TL burst fractures with a TLISC score ≤3 treated non-operatively were identified and included into this retrospective study. Age, sex, pain intensity, interpedicular distance (IPD), canal compromise, loss of vertebral body height and kyphotic angle (KA) were selected as potential risk factors and compared between the non-operative success group and the non-operative failure group.

**Results:**

One hundred and four patients successfully completed non-operative treatment, the other 25 patients were converted to surgical treatment because of persistent local back pain or progressive neurological deficits during follow-up. Our results showed that age, visual analogue scale (VAS) score and IPD, KA were significantly different between the two groups. Furthermore, regression analysis indicated that VAS score and IPD could be considered as significant predictors for the failure of conservative treatment.

**Conclusion:**

The recommendation of non-operative treatment for TLICS score ≤3 has limitations in some patients, and VAS score and IPD could be considered as risk factors for the failure of conservative treatment. Thus, conservative treatment should be decided with caution in patients with greater VAS scores or IPD. If non-operative management is decided, a close follow-up is necessary.

## Introduction

Spinal injuries in the thoracolumbar (TL) area are common on account of its location on a junction of spinal biomechanics, furthermore, up to 20% of all TL injuries are burst fractures[1]. Given this substantial morbidity and mortality, various recommendations for medical decision-making have been made for the treatment of TL trauma. However, the management of TL burst fracutres is still a matter of debate, with the treatment of stable TL burst fractures being the most controversial [2,3].

To develop an algorithm to guide the clincial decision between operative treatment and conservative treatment, the Spine Trauma Study Group proposed the TLICS system [4] (Table 1). Using a numerical scoring system derived from the injury morphology, posterior ligamentous complex (PLC) integrity, and neurological status, the TLICS allows for an algorithmic approach to the treatment of TL burst fracture. It is the first quantitative scoring system that can be used as a practical algorithm to orient the clinical decision-making between conservative and surgical management, and some reports have shown this classification to be both valid and reproducible [5–7].

**Table 1:** Thoracolumbar Injury Classifciation and Severity Score (TLICS).

**Injury morphology**	
**Qualifiers**	**Points**
Compression	1
Burst	+1
Translational/rotational	3
Distraction	4
**Neurological injury**	
**Qualifiers**	**Points**
Intact	0
Nerve root	2
Cord, conus medullaris	2 (complete)
	3 (incomplete)
Cauda equina	3
**Integrity of PLC**	
**Qualifiers**	**Points**
Intact	0
Suspected/indeterminate	2
Injured	3

Total score ≤3 for nonoperative intervention, 4 for nonoperative or operative intervention, and ≥5 for operative intervention.

In the TLICS classification, patients with a final score of ≤3 are indicated for nonoperative treatment. However, Mattei and colleagues reported a clinical case of comminuted burst fractures with a TLICS score of 2, in which the patient presented progressive kyphotic deformity by non-operative treatment[8]. This raised the question of the drawbacks and limitations of the TLICS[9]. Thus, it still remains a challenge to properly select treatment options for patients with a low TLICS score. The aim of this study is to identify several risk factors for the failure of conservative treatment of TL burst fractures based on the TLICS algorithm by using a retrospective case series.

## Patients and Methods

Patients with acute TL burst fractures and treated nonoperatively were retrospectively collected at the department of orthopaedics and rehabilitation of our hospital from June 2008 to December 2013. Written informed consent was conventionally signed by all patients before treatment, and patients’ private information was anonymized and de-identified before analysis. The research approach was approved by the ethics committee of Chongqing Medical University.

The clinical data were acquired by using plain radiographs, computed tomography (CT), and magnetic resonance images (MRI) preoperatively, together with information on age, medical history, injury mechanism, and clinical examination at the time of evaluation. In addition to the medical records, the above information was comprehensively reviewed and all severity scores were evaluated according to the TLICS algorithm, of which posterior ligamentous complex (PLC) status was assessed by short-tau inversion-recovery (STIR) sequences MRI[10]. Patients with scores less than 4 were included. The other inclusion criteria are as follows: a history of a definite lesion; the fractures were located within the TL region (T10 to L2), and involved the anterior and middle columns, with retropulsion of posterior bone fragments into the spinal canal as described by Denis[11]; presentation within 3 weeks of the time of injury; and no new neurological deficit. On the other hand, the exclusion criteria were as follows: major fractures at other sites, serious injuries associated with other major organ(s), pathological vertebral body collapse (e.g., tumour, tuberculosis, or osteoporosis), and preexisting spinal deformity and poor condition.

The nonoperative treatment scheme consists of pain control, venous thromboembolism prophylaxis, and immobilization. The patients were initially managed with 3–5 days of strict bed rest until they were fitted with a thoracolumbosacral orthosis (TLSO). Then, they were allowed to leave the bed while wearing the brace. The TLSO was required to be worn at all times except when the patient is lying flat in bed. All patients were instructed to wear the TLSO for 12 weeks and to begin weaning from it at the 8th week of the follow-up.

Because of individual differences, we defined failure of nonoperative treatment as the need for surgery because of persistent local back pain or progressive neurological deficits during follow-up. Operative interventions were managed with subtotal vertebraectomy for direct decompression reduction, reconstruction of the anterior and middle columns with a titanium cage or autologous bone grafting, and internal fixation through the anterior or posterior approach.

**Fig 1:**
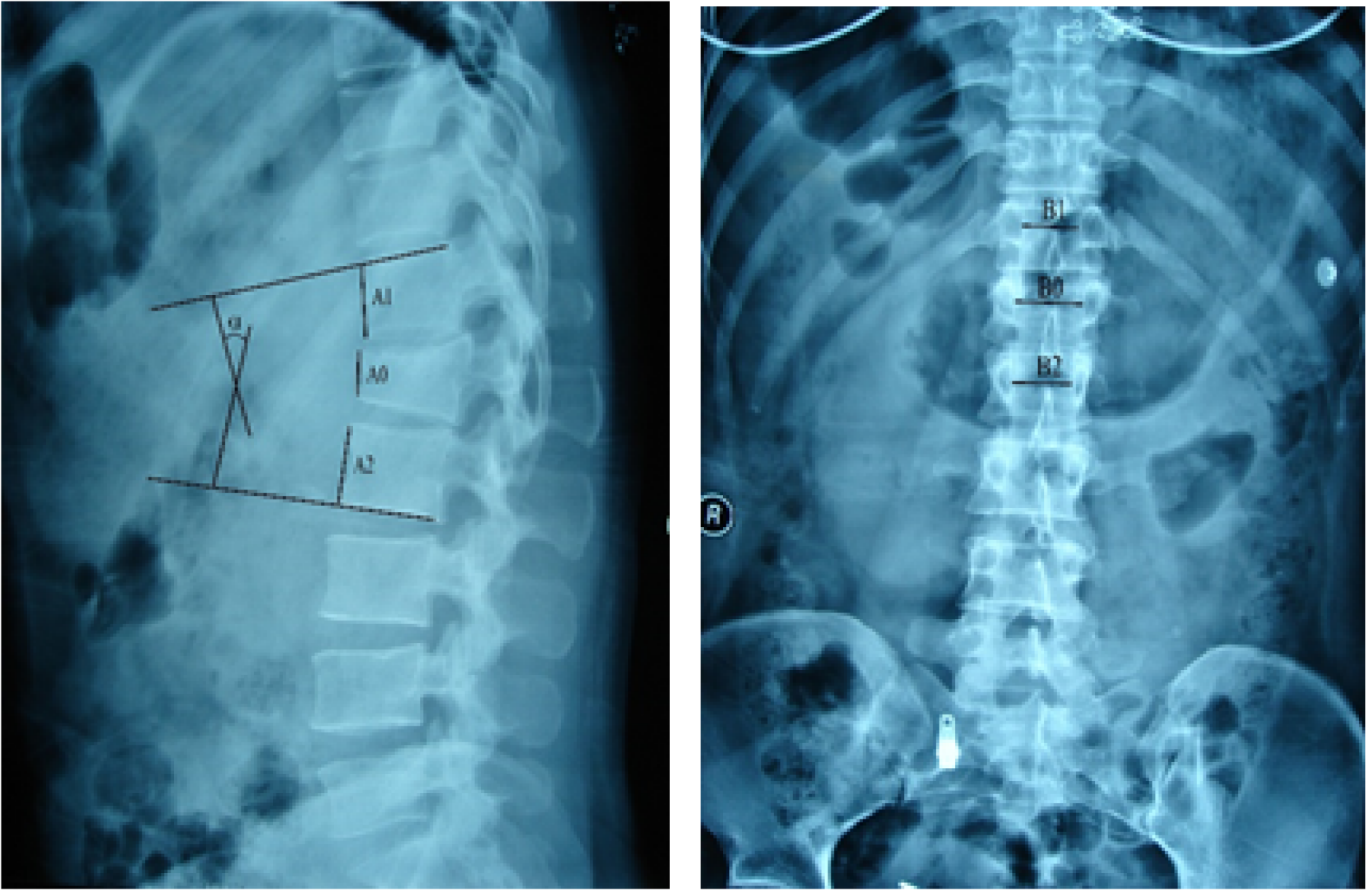
The kyphotic angle (KA) is measured by using the Cobb angle between the superior endplate of the intact vertebra above and the inferior endplate of the intact vertebra below. The percentages of loss of vertebral body height (LOVBH) and interpedicular distance (IPD) are measured by comparing the anterior body height and IPD with the mean of similar values obtained from the vertebrae immediately above and below. KA = Cobb angle; LOVBH = [(A1 + A2)- 2A0 / (A1 + A2)] × 100%; IPD = [2B0 - (B1 + B2) / (B1 + B2)] × 100%.

**Fig 2:**
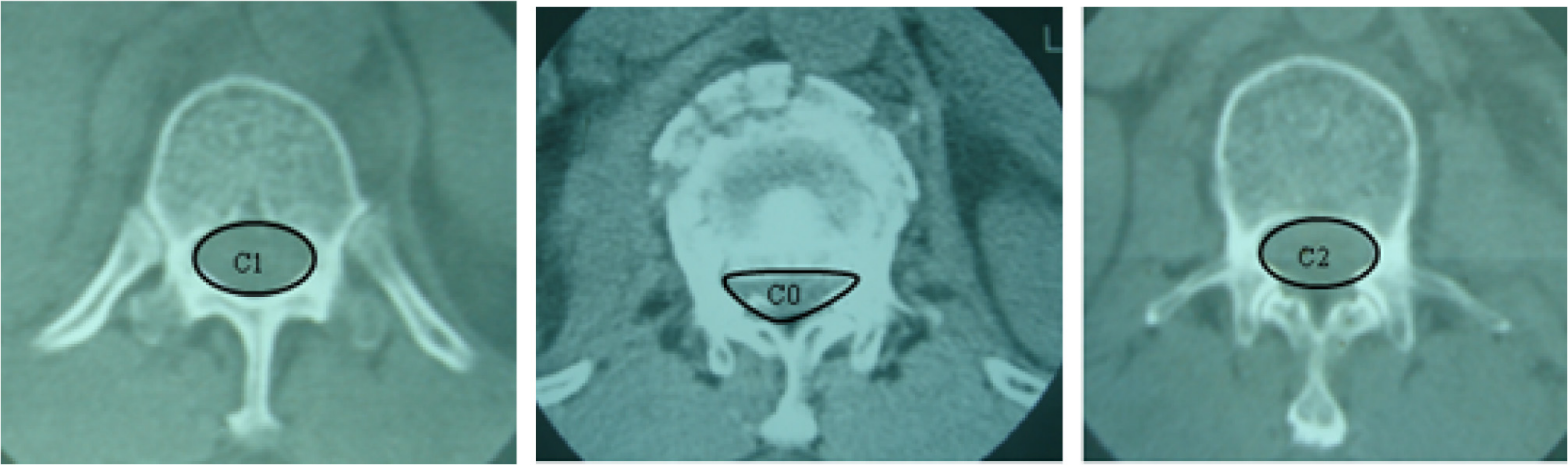
Canal compromise (CC) is calculated as a ratio of the canal area of the injured level to the average of that of the two adjacent uninjured segments. CC = [(C1 + C2)- 2C0 / (C1 + C2)] × 100%.

Age, sex, pain intensity, interpedicular distance (IPD), canal compromise (CC), loss of vertebral body height (LOVBD), and kyphotic angle (KA) were selected as potential risk factors and compared between the nonoperative success group and the nonoperative failure group. Pain intensity was measured on a 10-cm visual analogue scale (VAS). Anterior body height, IPD, and Cobb angle (KA) were directly measured on a plain radiographic film, and the percentages of IPD and LOVBH were calculated by using the formulas given in Fig 1. CC was evaluated by using CT axial images with AutoCAD software (Autodesk, USA), as shown in Fig 2.

Statistical analysis was performed by using SPSS software (version 19.0). Datas are presented as median ± standard deviation. Student’s t test for continuous data and the χ^2^ test for categorical data were used for comparisons of variables between the two groups. Binary logistic regression analysis was conducted to test the odds ratio and to select the factors that predict failure of conservative treatment. The level of significance was set at P < 0.05.

## Results

In total, 129 patients were identified and included into this study, including 90 men and 39 women, with an average age of 38.5 ± 9.1 years (range, 22–61 years). All of the patients had single-level fractures (18 at T10, 27 at T11, 39 at T12, 33 at L1, and 12 at L2), and the most common mechanism of fracture was a fall from height (63 patients), followed by traffic accidents (38 patients), blunt trauma (25 patients), and sports (3 patients). The average duration of follow-up was 36.5 ± 11.0 months (range, 12–66 months).

Ultimately, 104 patients successfully completed nonoperative management, comprising 72 men and 32 women, with ages of 37.1 ± 8.2 years. The mean follow-up time was 36.0 ± 10.2 months. The other 25 patients failed conservative treatment and had to receive surgery (18 men and 7 women, age 44.2 ± 10.8 years). The average duration of follow-up was 38.8 ± 13.9 months, and the operations were carried out at 4.8 ± 2.7 months after the injury. All patients who received surgical treatment had remission of clinical symptoms, except for one patient with internal fixation failure. On admission, the median pain score as measured by using VAS was 3.6 ± 1.3 for those with successful nonoperative treatment and 6.5 ± 1.9 for those with failed nonoperative treatment. The difference of sex and follow-up time between the nonoperative and operative groups did not show significance (P = 0.787 and P = 0.254, respectively), but was statistically significant for age and VAS (both P < 0.001) (Table 2).

**Table 2:** Patient Demographic Data.

	Nonoperative group	Operative group	P value
**No. of patients**	104	25	
**Sex** ^**b**^			0.787
Male	72	18	
Female	32	7	
**Age** ^**a**^	37.1 ± 8.2	44.2 ± 10.8	<0.001
**Level of fracture**			
T10	14	4	
T11	21	6	
T12	32	7	
L1	27	6	
L2	10	2	
**Mechanism of injury**			
Fall from height	52	11	
Traffic accidents	31	7	
Blunt trauma	18	7	
Sports	1	2	
**VAS score** ^**a**^	3.6 ± 1.3	6.5 ± 1.9	<0.001
**Follow-up (months)** ^**b**^	36.0 ± 10.2	38.8 ± 13.9	0.254

Data are presented median ± standard deviation.

^a^Significant difference between the nonoperative and operative groups.

^b^No significant difference between the nonoperative and operative groups.

Radiologic analysis indicated that only IPD and KA showed significant differences between the nonoperative and operative groups. The IPD in the nonoperative group was 11.2 ± 6.6%, which was significantly less than that in the operative group (29.7 ± 5.5%, P < 0.001). Meanwhile, the KA values in the nonoperative and operative groups were 11.3 ± 4.2° and 22.7 ± 8.3°, respectively, with the difference attaining statistical significance (P = 0.012). However, the LOVBH and CC in the nonoperative group (29.4 ± 7.7% and 25.3 ± 8.7%, respectively) were lower than that in the operative group (31.5 ± 7.3% and 28.0 ± 9.7%), but the difference was not significant (P = 0.222 and 0.165, respectively) (Table 3).

**Table 3:** Radiologic Results Analysis.

	Nonoperative	Operative	P value
**Interpedicular distance** ^**a**^ **(IPD)**	11.2 ± 6.6%	29.7 ± 5.5%	<0.001
**Canal compromise** ^**b**^ **(CC)**	25.3 ± 8.7%	28.0 ± 9.7%	0.165
**Loss of vertebral body height** ^**b**^ **(LOVBH)**	29.4 ± 7.7%	31.5 ± 7.3%	0.222
**Kyphotic angle** ^**a**^ **(KA)**	11.3 ± 4.2°	22.7 ± 8.3°	0.012

Data are presented as median ± standard deviation.

^a^Significant difference between the nonoperative and operative groups.

^b^No significant difference between the nonoperative and operative groups.

Furthermore, binary logistic regression analysis was performed for age, VAS score, IPD, and KA. The results showed that only IPD (odds ratio [OR], 1.504; 95% confidence interval [95% CI], 1.099–2.058; P = 0.011 < 0.05) and VAS score (OR, 2.981; 95% CI, 1.103–8.059; P = 0.031 < 0.05) could be considered significant risk factors for failure of nonoperative treatment, necessitating a close follow-up after conservative treatment is decided. Although age (OR, 1.140; 95% CI, 0.991–1.312; P = 0.068 > 0.05) and KA (OR, 1.205; 95% CI, 0.965–1.504; P = 0.099) in the operative group was much greater than that in the nonoperative group, the regression model demonstrated that age and KA were just approximately (but still nonstatistically) predictors for failure of nonoperative treatment (Table 4).

**Table 4:** Binary Logistic Regression Analysis.

	Odds ratio	95% CI	P value
**Age** ^**a**^	1.140	0.991 1.312	0.068
**VAS score** ^**b**^	2.981	1.103 8.059	0.031
**IPD** ^**b**^	1.504	1.099 2.058	0.011
**KA** ^**a**^	1.205	0.965 1.504	0.099

^a^No significant difference between the nonoperative and operative groups.

^b^Significant difference between the nonoperative and operative groups.

## Discussion

The most important finding of this study was that the parameters VAS score and IPD could be considered as risk factors for the failure of conservative treatment of TL burst fractures according to TLICS less than 4. The original intention of this study is that we find it is surprising given the clear regional variations in treatment practices for TL burst fractures[12–14]. The TLICS system introduced by Vaccaro seems like a simple and reproducible severity scoring system to help therapeutic decision making, however, most of the studies on the clinical reliability and validity of the TLICS system have been limited to small case series or reported by the developers of the TLICS system themselves[6,7,15,16]. The controversy about using the TLICS to guide treatment decision making has been increasing gradually. The results of this study confirmed that a total of 129 patients with a TLICS score less than 4 were retrospectively identified and evaluated, showing that 25 patients (up to 19.4%) without neurological dysfunction and PLC damage still failed nonoperative treatment and ultimately required surgery.

A consensus has emerged that surgical treatment needs for severe unstable spinal injuries with neurological damage or clear lesions of the PLC. However, TL burst traumas with TLICS score ≤3 are hardly accompanied by PLC damage or neurological deficits (otherwise, a score of at least ≥4 is given),and the treatment for these stable burst fractures is more controversial. A retrospective study including 458 patients illustrated that up to 53.4% patients fail to match the TLICS score for surgical treatment, and all of the patients in this subgroup had burst fractures without neurological deficits[17]. Wood et al. reported a randomized prospective trial comparing spinal fusion to nonoperative treatment with follow-up at 16 to 22 years, whose results showed no clinically significant difference between the two groups[3]. Other data is just contradictory to the conclusion of Wood’s. Siebenga et al.[2] reported that 34 patients with a type A3 TL fracture were randomized for operative and nonoperative treatment after a mean follow-up of 4.3 years. At the end of follow-up, both radiological and clinical functional outcomes were significantly better in the operative group.

In our study, the results showed that patients who failed conservative treatment as recommended by TLICS had an average age of 44.2 ± 10.8 years, which is significantly biger than the age of 37.1 ± 8.2 years in the group who successfully completed nonoperative treatment. These results can be partly explained by the fact that the bone quality and bone regeneration ability are worse in older than in younger patients. Concerning pain outcomes, our findings suggest that those treated nonoperatively reported a lower degree of VAS score on admission. The VAS score is an important self-assessment for evaluating pain intensity. Previous studies had always focused on the VAS outcomes at follow-up to compare the curative effect between nonoperative and operative treatment[18–20]; our results indicate that the higher the degree of pain patients feel after injury, the poorer the outcome of nonoperative treatment will be.

Radiographic examination revealed that the IPD and KA in the operative group increased significantly compared with that in the nonoperative group. The widening of the distance between the pedicles, after the spine received the axial trauma, allows posterior bone fragments to move into the spinal canal, which may lead to neurological compression. A retrospective analysis of 260 patients illustrated that the IPD proved to be a reliable parameter to assess narrowing of the spinal canal, neurological deficits, and laminar fractures[21], which suggests that IPD may be an important parameter for evaluating the severity of TL burst fractures. KA is an interesting parameter in this study, of which patients went on to have surgery had kyphosis of > 23° compared to 11° in the successfully nonoperative group. However, a biomechnical study has reported that kyphotic angle >20° implied disruption of PLC[22]. Then, the patients in operative group should have had TLICS of at least 4, was the TLICS not applied correctly in this study? We don’t think so. Radcliff et al. directly reported that kyphosis >20° is not predictive of PLC injury in patients with TL burst fractures[23]. Another study by Petersilge et al. did not find any significant correlation between the radiographic appearance of the fracture and PLC injury[24]. In addition, recent studies have confirmed that bony parameters, including loss of vertebral height and kyphosis, do not correlate with clincial outcomes[2,25]. This in turn raises a question that if greater KA means PLC injury, further confirming spinal instability, then why it is not associated with the outcome of TL burst fracture treatment? By the further logistic regression analysis, we also found that KA is not related with failure of nonoperative treatment. These studies demonstrated that the radiogrphical parameter such as KA is not associated with PLC injury. On the other side, MRI is currently considered the imagingg “gold standard” for determination of PLC injury, because of its high sensitivity and specificity[10,26]. In the present study, we used the STIR-weighted MRI to directly indentify the PLC status, rather than indirectly indentifing by the bone parameters.

Furthermore, a binary logistic regression analysis for age, VAS score, IPD, and KA showed that only VAS score and IPD were significant risk factors of failure of nonoperative treatment, indicating the requirement for surgical intervention in the future. However, the present study has limitations related to the initial trial design, such as its retrospective nature, the limited sample size, and the absence of randomization between the operative and nonoperative treatment groups. Nevertheless, our results still provided some important red flags concerning the possible risk factors of such fractures that were not properly identified by the TLICS.

## Conclusion

The TLICS algorithm focuses on three important aspects of TL burst fractures and may therefore be useful to decide whether to perform conservative treatment or surgery according to the final score. However, the nonoperative treatment recommendation for a TLICS score of ≤3 has limitations in some patients who may need to receive operative treatment in the future because of a progressive symptomatology. The present study advocates that the VAS score and IPD parameters could be considered as risk factors for the failure of conservative treatment. Therefore, conservative treatment should be decided with caution in patients with greater VAS scores or IPD. If nonoperative management is decided, a close follow-up is necessary.
